# Expression analysis of limb element markers during mouse embryonic development

**DOI:** 10.1002/dvdy.24671

**Published:** 2018-11-07

**Authors:** Alexandra Rafipay, Amanda L. R. Berg, Lynda Erskine, Neil Vargesson

**Affiliations:** ^1^ School of Medicine, Medical Sciences and Nutrition, Institute of Medical Sciences University of Aberdeen Foresterhill Aberdeen

**Keywords:** Gene expression, limb development, forelimb, hindlimb, vascular, cartilage, nerve, muscle, tendon, joint, Alcian Blue/Alizarin Red

## Abstract

Background: While data regarding expression of limb element and tissue markers during normal mouse limb development exist, few studies show expression patterns in upper and lower limbs throughout key limb development stages. A comparison to normal developmental events is essential when analyzing development of the limb in mutant mice models. Results: Expression patterns of the joint marker *Gdf5*, tendon and ligament marker *Scleraxis*, early muscle marker *MyoD1*, and blood vessel marker *Cadherin5 (Cdh5)* are presented during the most active phases of embryonic mouse limb patterning. Anti‐neurofilament staining of developing nerves in the fore‐ and hindlimbs and cartilage formation and progression also are described. Conclusions: This study demonstrates and describes a range of key morphological markers and methods that together can be used to assess normal and abnormal limb development. *Developmental Dynamics* 247:1217–1226, 2018. © 2018 The Authors. *Developmental Dynamics* published by Wiley Periodicals, Inc. on behalf of American Association of Anatomists

## Key Findings

- Expression patterns of molecular markers throughout both fore‐ and hindlimb development ‐ which can be used to assess normal and abnormal development.
- Detailled description of innervation during fore‐ and hindlimb development confirming innervation first seen after limb patterning events have begun.
- Description of cartilage development and progression indicates alizarin red staining not seen until E15.5 in both fore‐ and hindlimbs.
- Hindlimb lags behind forelimb molecularly and morphologically until E14.5.
- Detailled description of methods used to study fore‐ and hindlimb development.

## Introduction

Although significant progress has been made in understanding the molecular and morphological mechanisms controlling limb development, just how neuronal innervation, cartilage formation and progression, vascular patterning, tendon development, and joint development are so intricately controlled and regulated is still unclear. Analysis of genetic mouse mutants is frequently used to identify factors essential for normal limb development and their mechanism of action, and requires a comparison to be made to normal development of limb elements.

C57BL/6 mouse embryos develop in 18.5 days, and the forelimb bud is apparent from embryonic day (E) 9.5. The hindlimb consistently lags about a half‐day behind forelimb development (Martin 1990). The limb develops in a proximodistal manner, with the proximal stylopod developing before the zeugopod and distal autopod (Muneoka et al., 1989; Martin 1990). Muscle precursors migrate in to the forelimb around E10.5, and expression of myogenic factors is detected in the limb at E11, with clear muscle blocks seen at E12.5 (Sassoon et al., 1989; Martin 1990). Nerve fascicles, originating from the brachial plexus and the spinal lumbosacral plexus, are present in the proximal mouse forelimb and hindlimb, respectively, at E11.5 (Martin 1990). Tendon progenitor cells in the limb condense and differentiate into tenocytes, with pre‐tendon mesenchymal masses detected at E13.5 (Perez et al., 2003; Birch et al., 2013). Tenocytes then produce extracellular matrix molecules, including type I and III collagen, and organize them into tendon fibrils (Birch et al., 2013; Liu et al., 2015). Fibrils become further organized into tendon fibers, and by E14.5 tendons are visible in the digits (Perez et al., 2003). By E14.5, distal muscles are also visible, the nerves have formed dense plexuses, and the epidermis is thickening. Skeletal patterning of the limbs begins with the formation of cartilage condensations, from around E11.5 (Martin, 1990), which are the precursors of bones of the limbs (Martin, 1990; Nowlan et al., 2010). These elements form in a proximal to distal manner: For example, the humerus/femur forms before the radius and ulna/tibia and fibula, which form before the digits (Martin, 1990; Vargesson and Hootnick, 2017). Joint development begins with the formation of the interzone, a region of flat, condensed mesenchymal cells. The formation of the interzone is followed by joint cavitation. Cavitation of mouse proximal limb joints is visible at E15.5 (Mitrovic 1977; Wang et al., 2001). Limb development is typically complete around E18.5 (Martin 1990).

We have performed a detailed spatiotemporal analysis of markers of joint (*Gdf5*), tendon (*Scleraxis)*, muscle (*MyoD1*), vascular (*Cdh5)*, neuronal (anti‐neurofilament), and cartilage (Alcian Blue/Alizarin Red staining) development. Although aspects of the expression patterns of these genes and tissue markers are published, single studies showing expression patterns of all of these markers in both upper and lower limbs are lacking, and there is limited analysis of the temporal changes in expression throughout limb development. Moreover, for several of these markers, distinct aspects of the expression patterns are reported in multiple publications, making determination of the spatiotemporal changes in expression difficult to ascertain.

Distinct aspects of *Gdf5* expression have been reported in several publications that combined have shown *Gdf5* expression between E11.5 and E14.5 in the presumptive sites of joint development and the forming joint interzone (Chen et al., 2016; Degenkolbe et al., 2013; Hellman et al., 2012; Houweling et al., 2001; Huang et al., 2016; Meech et al., 2005; Ota et al., 2007; Perez et al., 2010; Seemann et al., 2005; Sohaskey et al., 2008). Uniquely, we have shown side‐by‐side *Gdf5* expression across a range of key stages of development in both the forelimb and hindlimb. *MyoD1* expression, a myogenic helix‐loop‐helix protein family restricted to differentiating skeletal muscle (reviewed in Tapscott, 2005), has been examined in the context of comparison to mutant models but infrequently as a marker of muscle development alone (Grifone et al., 2005; Kablar et al., 1997; Laclef et al., 2003; L'honoré et al., 2010; Mayeuf‐Louchart et al., 2016), highlighting the requirement for a summative analysis of expression during mouse limb development. *Scleraxis*, a basic helix‐loop‐helix factor expressed in connective tissues that form muscle attachments (Schweitzer et al., 2001), is reexamined here alongside other limb element development markers to allow a comparison to the development of the other tissues (Murchison et al., 2007; Schweitzer et al., 2001). *Cdh5* expression, an endothelial‐specific cadherin expressed during early vasculogenesis with expression maintained throughout adult life (Neuhaus et al., 2014), has not, as far as we are aware, been studied throughout mouse limb development, and indeed whole‐mount in situ analysis of blood vessel markers is rare, with the majority of studies describing expression at one or two time points as a comparison to mutant models (Eshkar‐Oren et al., 2009; Eshkar‐Oren et al., 2015; Ota et al., 2007; Vieira et al., 2007). Nerve patterns are usually assessed at specific time points to see the effect of a mutation or morphological interaction. Here, we show nerve innervation patterns throughout limb outgrowth. Similarly, cartilage and bone analysis is usually assessed in mouse limb studies at specific time points to study specific gene function and action (for example, Amarilio et al., 2007; Collinson et al., 2018; Firulli et al., 2005; Nowlan et al., 2010; Ray et al., 2015; Tavella et al., 2004). Here, we show in detail the progression of cartilage and bone development throughout fore‐ and hindlimb patterning. Taken together, our description of the spatiotemporal expression of these markers can be used as a toolbox to assess the formation and development of key tissues in normal and abnormal limb development.

## Results

We have analyzed the dynamic expression in mouse fore‐ and hindlimbs of *Gdf5, MyoD1*, and *Cdh5* from E13.5 to E15.5; *Scleraxis* from E12.5 to E15.5; anti‐neurofilament from E10.5 to E14.5; and cartilage/bone development and progression from E11.5 to E16.5. Although mouse limb development begins around E9.0, the limb bud at this stage consists of a mass of undifferentiated cells, and the main limb structures—including tendons, skeleton, muscles, and vessels—are organized between E12.5 and E15.5 (Bénazet and Zeller 2009; Marcon et al., 2011; Martin, 1990; Tickle 2006).

### Expression Pattern of the Joint Marker *Gdf5*

**Figure 1:**
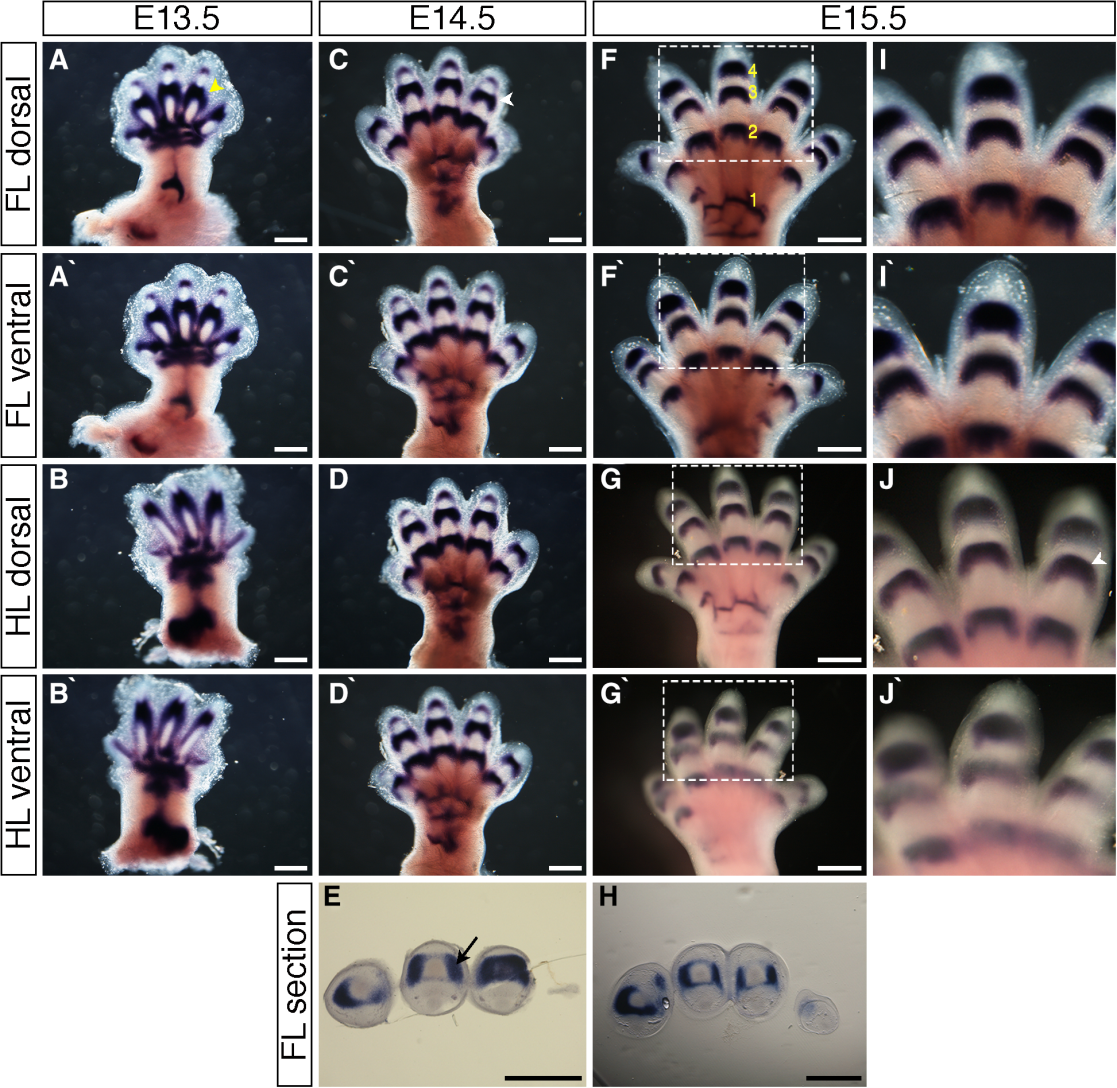
Expression patterns of *Gdf5* during mouse limb development. **A–D`,F–G`,I–J`**: Whole‐mount in situ hybridization for *Gdf5* in E13.5 (A–B`), E14.5 (C–D`), and E15.5 (F–G`) dorsal forelimbs (A,C,F), ventral forelimbs (A`,C`,F`), dorsal hindlimbs (B,D,G), and ventral hindlimbs (B`,D`,G`). I`,J`: Higher‐magnification images of regions highlighted in white dashed boxes in F–G`, respectively. **E,H**: Sections through distal interphalangeal joints of E14.5 (E) and E15.5 (H) forelimbs stained by in situ hybridization for *Gdf5*; dorsal at top, anterior to right. *Gdf5* is expressed in prechondrogenic condensations at E13.5 (A yellow arrowhead), and in interphalangeal, metacarpophalangeal, and carpometacarpal joints at E14.5 and E15.5 (C,J white arrowhead), and in joint interzones (E black arrow). Developing joints are numbered (F yellow numbers) 1: carpometacarpal joint; 2: metacarpophalangeal joint; 3: proximal interphalangeal joint; 4: distal interphalangeal joint. Scale bars = 500 µm.

Cartilage elements develop in a proximodistal fashion, with the humerus/femur forming before the digits. Digits first develop as “rays” (digit and metacarpal/metatarsal as a continuous unit with webbing between the digits proper), and at E13.5, *Gdf5* was expressed along rays in presumptive joint regions that develop to separate rays into proximal, intermediate, and distal phalanges (Fig. 1A yellow arrowhead, Fig. 1A–B`). By E14.5, there was no longer continuous *Gdf5* expression along cartilage condensations, and thick bands of *Gdf5* expression were visible in carpometacarpal/tarsometatarsal joints, metacarpophalangeal/metatarsophalangeal joints, and the developing distal interphalangeal joint in joint interzones (Fig. 1C white arrowhead, Fig. 1C`–E). At E15.5, joint patterning became more apparent and *Gdf5* was expressed as thinner bands in the more defined carpometacarpal/tarsometatarsal joints, metacarpophalangeal/metatarsophalangeal joints, and proximal and distal interphalangeal joints. (Fig. 1F–J`). In sections through the joints, strong expression of *Gdf5* was detected in joint interzones (Fig. 1E black arrow, Fig. 1H).

### Expression Pattern of the Tendon and Ligament Marker *Scleraxis*

**Figure 2:**
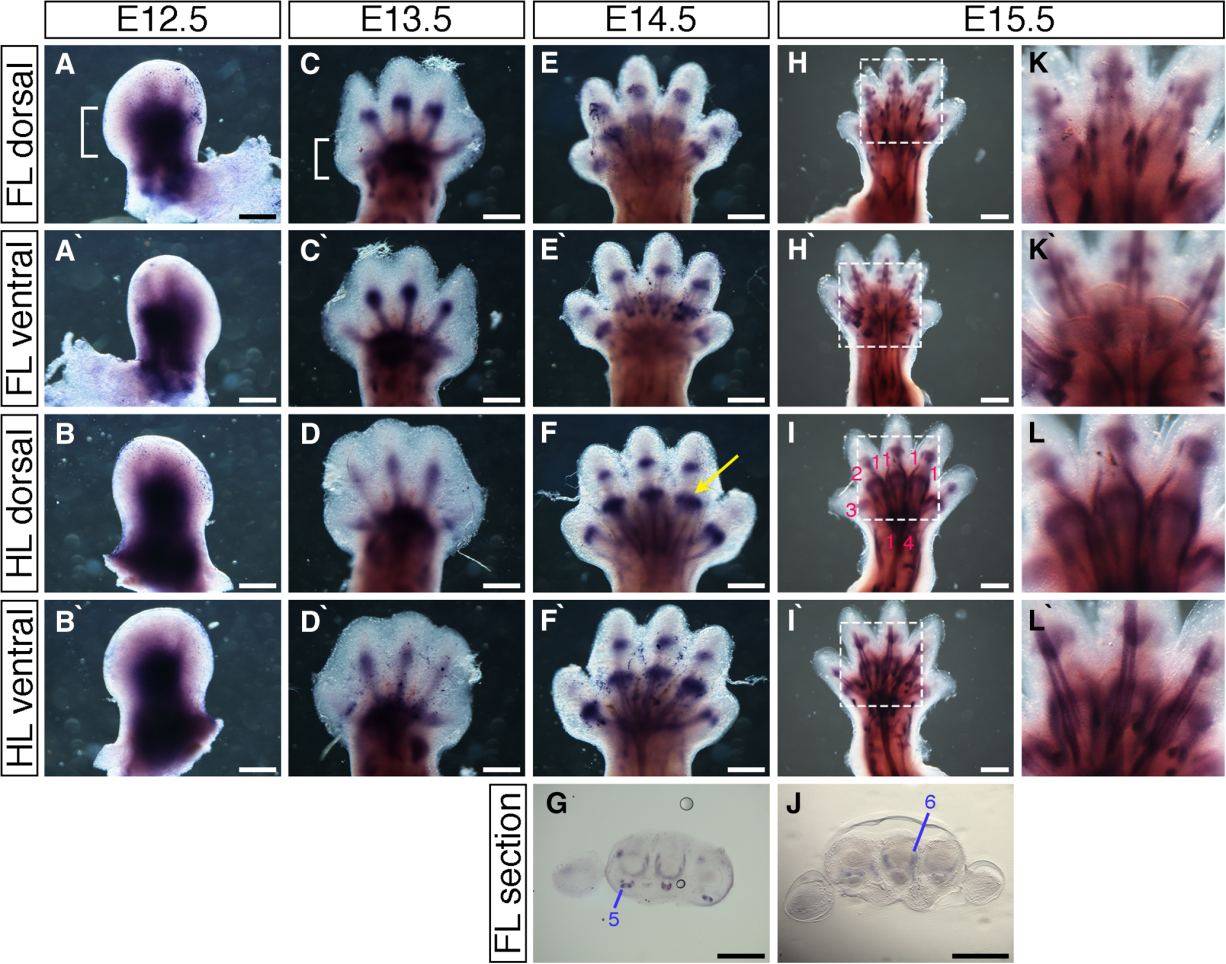
*Scleraxis* expression pattern during mouse limb development. **A–F`,H–I`,K–L`**: Whole‐mount in situ hybridization for *Scleraxis* at E12.5 (A–B`), E13.5 (C–D`), E14.5 (E–F`), and E15.5 (H–I`) in dorsal forelimbs (A,C,E,H), ventral forelimbs (A`,C`,E`,H`), dorsal hindlimbs (B,D,F,I), and ventral hindlimbs (B`,D`,F`,I`). K–L`: Higher magnification of regions highlighted in white dashed boxes in H–I`, respectively. **G,J**: Section images through proximal metacarpal joints at E14.5 (G) and E15.5 (J) of forelimbs stained by in situ hybridization for *Scleraxis*; dorsal at top, anterior to left. Blue lines indicate position of flexor tendons. Wrist tendon progenitors (A white bracket) at E12.5 give rise to zeugopod tendons (C white bracket). Phalangeal insert sites for connective tissues can be distinguished (F yellow arrow). Tendons are numbered (I pink numbers; G–J blue numbers) 1: extensor digitorium communis, 2: extensor carpi radialis longus, 3: extensor pollicis, 4: extensor carpi ulnaris, 5: flexor digitorum profundus, 6: lumbrical tendon. Scale bars = 500 µm.

Expression patterns of *Scleraxis* were determined between E12.5 and E15.5, since the vast majority of connective tissue patterning in the limbs occurs between these stages. At E12.5, *Scleraxis* was expressed broadly throughout the early autopod (hand‐/footplate) (Fig. 2A white bracket), with weak expression in the primitive digits (Fig. 2A–B`). At E13.5, *Scleraxis* was expressed in the hand‐ and footplate (Fig. 2C white bracket) and in the digit rays, with thicker regions of expression at presumptive joint sites (compare to Fig. 1A–B`). *Scleraxis* expression domains were wider at presumptive joint sites of the forelimb compared to the hindlimb (Fig. 2C–D`), comparable to more defined *Gdf5* expression at presumptive joint regions of the forelimb (Fig. 1A,A`). At E14.5, distinct bands of *Scleraxis* expression were visible through the autopod and extending to the digit tips. Expression was strongest at regions of joint development (Fig. 2F yellow arrow), where attachment sites between connective tissue and the skeleton form (Fig. 2E–F`). In E15.5 embryos, *Scleraxis* was expressed in distinct bands that extended along the digits, with strong areas of expression persisting around developing joint regions (Fig. 2H–I`,K–L`). Expression of *Scleraxis* corresponding to flexor tendons could be seen at the ventral side of section images (Fig. 2G,J, blue lines).

### Expression Pattern of the Muscle Marker *MyoD1*

**Figure 3:**
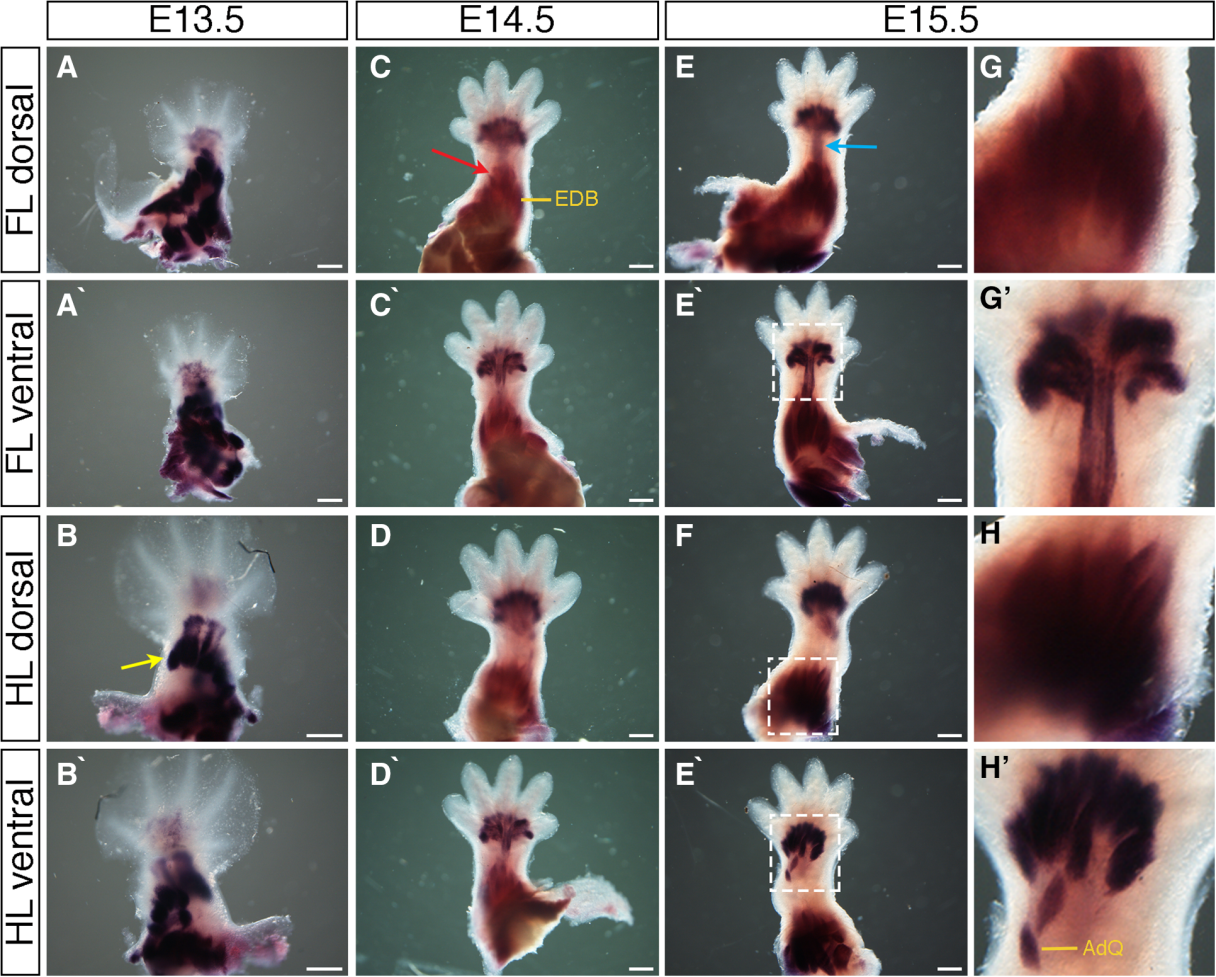
Expression of *MyoD1* during mouse limb development. **A–H`**: Whole‐mount in situ hybridization for *MyoD1* at E13.5 (A–B`), E14.5 (C–D`), and E15.5 (E–F`) in dorsal forelimbs (A,C,E), ventral forelimbs (A`,C`,E`), dorsal hindlimbs (B,D,F), and ventral hindlimbs (B`,D`,F`). G–H`: Higher magnification of regions highlighted in white dashed boxes in E–F`, respectively. Expression is seen in developing muscle blocks (B yellow arrow; C red arrow) and in a band connecting zeugopod and autopod muscle blocks in the forelimb of E15.5 embryos (E blue arrow). Some distinct muscles are highlighted (C, EDB: extensor digitorum brevis; H`, AdQ: abductor quinti muscle). Scale bars = 500 µm.

At E13.5, whole‐mount in situ hybridization analysis of *MyoD1* revealed developing muscle patterning in the fore‐ and hindlimb stylopod and zeugopod, with stronger staining in regions of developing muscle blocks (Fig. 3B yellow arrow). Faint expression was visible in the hand‐ and footplate (Fig. 3A–B`). At E14.5, *MyoD1* showed strong expression in the proximal zeugopod (forearm/leg), with stronger expression where bands of muscle are developing (Fig. 3 red arrow, Fig. 3C`), with stronger expression in the forelimb zeugopod compared to the hindlimb zeugopod. *MyoD1* was also expressed in the hand‐ and footplate, showing muscle bands from the center radiating to digit bases. A thin band of expression through the middle of the wrist connected expression regions of the zeugopod to the autopod in the forelimb, but no such expression band from the zeugopod to the autopod is seen in the hindlimb (Fig. 3C–D`). Expression at E15.5 was similar to that at E14.5 in the zeugopod and autopod. The band of expression connecting the two regions was stronger in the forelimb in E15.5 compared to E14.5 (Fig. 3E blue arrow, Fig.E`–H`). *MyoD1* expression in the limbs, when viewed at higher magnification, demonstrates the definition and organization of fibers into defined muscle sets by E15.5 (Fig. 3G–H`).

### Expression Pattern of the Endothelial Cadherin *Cdh5*

**Figure 4:**
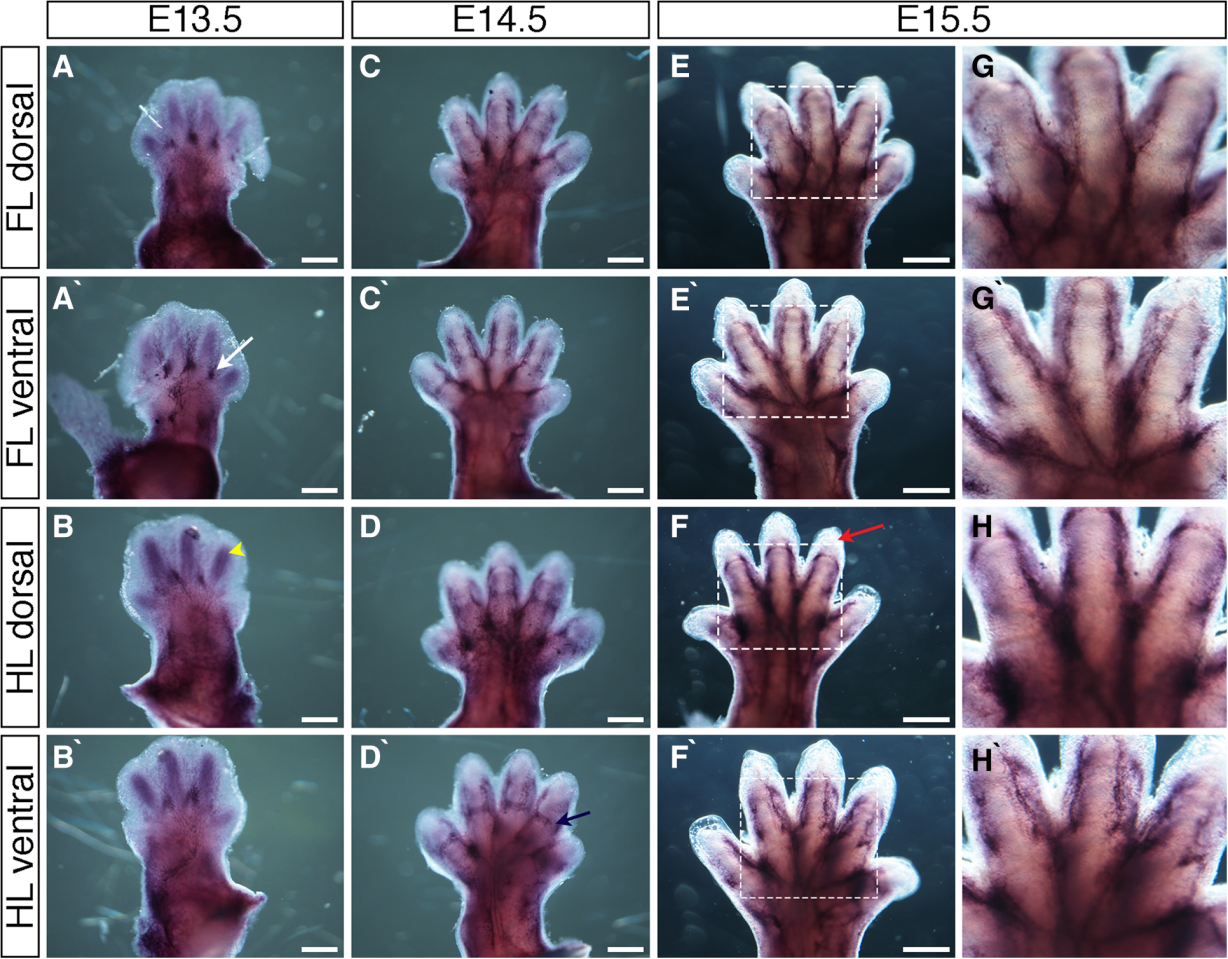
Expression of *Cadherin5 (Cdh5)* during mouse limb development. **A–H`**: Whole‐mount in situ hybridization for *Cdh5* at E13.5 (A–B`), E14.5 (C–D`), and E15.5 (E–F`) in dorsal forelimbs (A, C, E), ventral forelimbs (A`,C`,E`), dorsal hindlimbs (B,D,F), and ventral hindlimbs (B`,D`,F`). Strong regions of expression are seen at the digit base, between digits (A` white arrow). Blood vessels are visible in regions of developing cartilage at E13.5 (B yellow arrowhead). Bands of expression across presumptive joint sites are visible at E14.5 (D navy arrow). Avascular distal mesoderm is indicated (F` red arrow). G–H`: Higher magnification of regions highlighted in white dashed boxes in E–F`, respectively. Scale bars = 500 µm.

*Cdh5* expression was visible through the zeugopod and in the hand‐ and footplate at E13.5 and along the digit rays, but little expression was apparent interdigitally (Fig. 4A–B`). Stronger regions of expression existed in between digits at the digit base (Fig. 4A` white arrow). Larger vessels and more complex networks developed from these relatively simple early networks by E14.5. Thick bands of expression radiated from the hand‐ and footplate and along the digit borders. Expression was also visible at presumptive joint sites along the digits (Fig. 4C–D, Fig. 4D` blue arrow). At E15.5, strong bands of *Cdh5* expression existed along the wrist/ankle, into the hand‐ and footplate, and along digit borders. Bands of expression along the presumptive joint sites were no longer present (Fig. 4E–F`), confirmed when viewing the limbs at higher magnification (Fig. 4G–H`). At all ages, no expression was observed in the distal digit tips (Fig. 4E–F, Fig. 4F` red arrow).

### Neuronal Patterning in the Developing Mouse Limb

**Figure 5:**
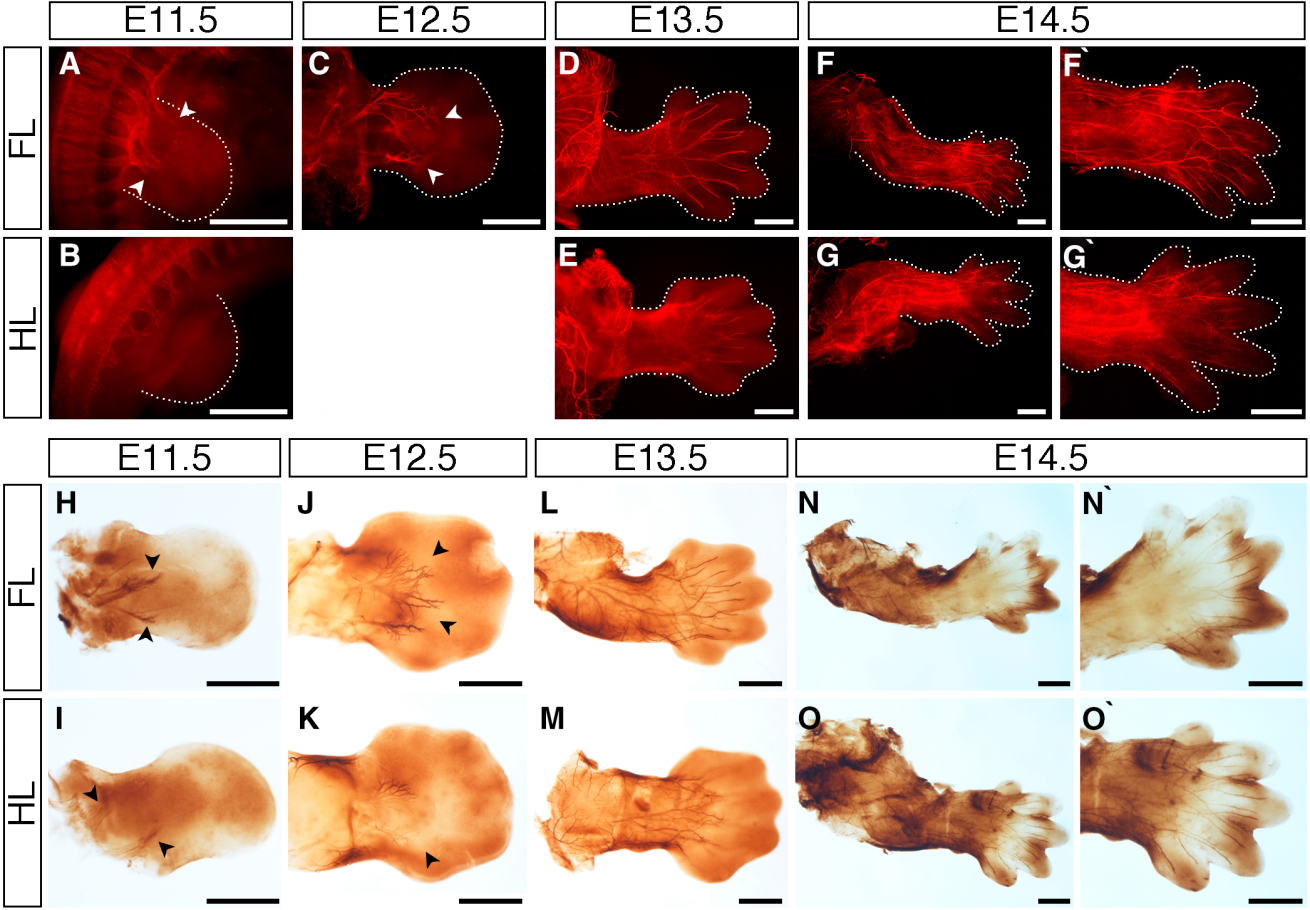
Development of limb innervation I. **A–O`**: Staining with an anti‐neurofilament antibody using a fluorescent secondary antibody (A–G’) or peroxidase conjugated secondary antibody (H–O`) to visualize limb innervation patterns in E11.5 (A,B,H,I), E12.5 (C,J,K), E13.5 (D,E,L,M), and E14.5 (F–G`,N–O`) forelimbs (A,C,D,F,F`,H,J,L,N,N`) and hindlimbs (B,E,G,G’,I,K,M,O,O`). Nerves enter the limb bud at E11.5 (A,B,H,I arrowheads) and handplate at E12.5 (C,J,K arrowheads). Innervation is more advanced in the forelimbs up to E13.5, where nerves have reached the digits in the forelimb (D,L) and remain at the base of the footplate in the hindlimb (E,M). Nerve innervation is throughout the limbs by E14.5 (F,G,N,O). High‐magnification images of the E14.5 forelimb handplate (F`,N`) and hindlimb footplate (G`,O`) show nerves have reached the tips of the condensing digits. Limbs are attached to the embryo body in A–E, and detached in F–O`. Dotted lines indicate the outline of the limbs in the fluorescent images. Scale bars = 500 µm.

Innervation of neuronal development in the mouse embryonic limb was examined using an anti‐neurofilament antibody between E10.5 and E14.5 by whole‐mount immunofluorescence (Fig. 5A–G`) and immunoperoxidase staining (Fig. 5H–O`). Both methods gave similar results in terms of neuronal innervation patterns. Forelimbs obtain innervation from spinal nerves via the brachial plexus, and hindlimbs from spinal nerves via the lumbar plexus. At E10.5, no nerves were present in the early fore‐ or hindlimb buds (data not shown). The first signs of limb innervation from spinal nerves were evident in the E11.5 forelimb (Fig. 5A,H arrowheads). In the hindlimb, innervation was more variable, where it was evident in some limbs (Fig. 5I arrowheads) but not others (Fig. 5B). This likely reflects differences in the precise age of the embryos, which can vary up to 0.5 day between embryos of a single litter. However, if present, nerves in the E11.5 hindlimb always extended for a shorter distance into the limb compared to the forelimb (Fig. 5H,I). Innervation of forelimbs and hindlimbs then continues at a rapid pace; however, innervation in the hindlimb lags behind that in the forelimb until E14.5 (Fig. 5A–0`). Main nerve trunks were present at E12.5, including branches that are targeted to proximal tissues in both forelimbs and hindlimbs (Fig. 5C,J,K). By E13.5, more distal nerve branches could be seen, proximal patterning of nerves had become more intricate, and nerves had reached the hand‐ and footplate (Fig. 5D,E,L,M). By E14.5, nerves had reached digit tips in both forelimbs and hindlimbs, thicker nerve branches were visible, and patterning was substantially more complex (Fig. 5F,G,N,O), further confirmed when looking at the hand‐ and footplates in higher magnification (Fig. 5F`,G`,N`,O`).

### Cartilage Formation and Progression in the Developing Mouse Limb

**Figure 6:**
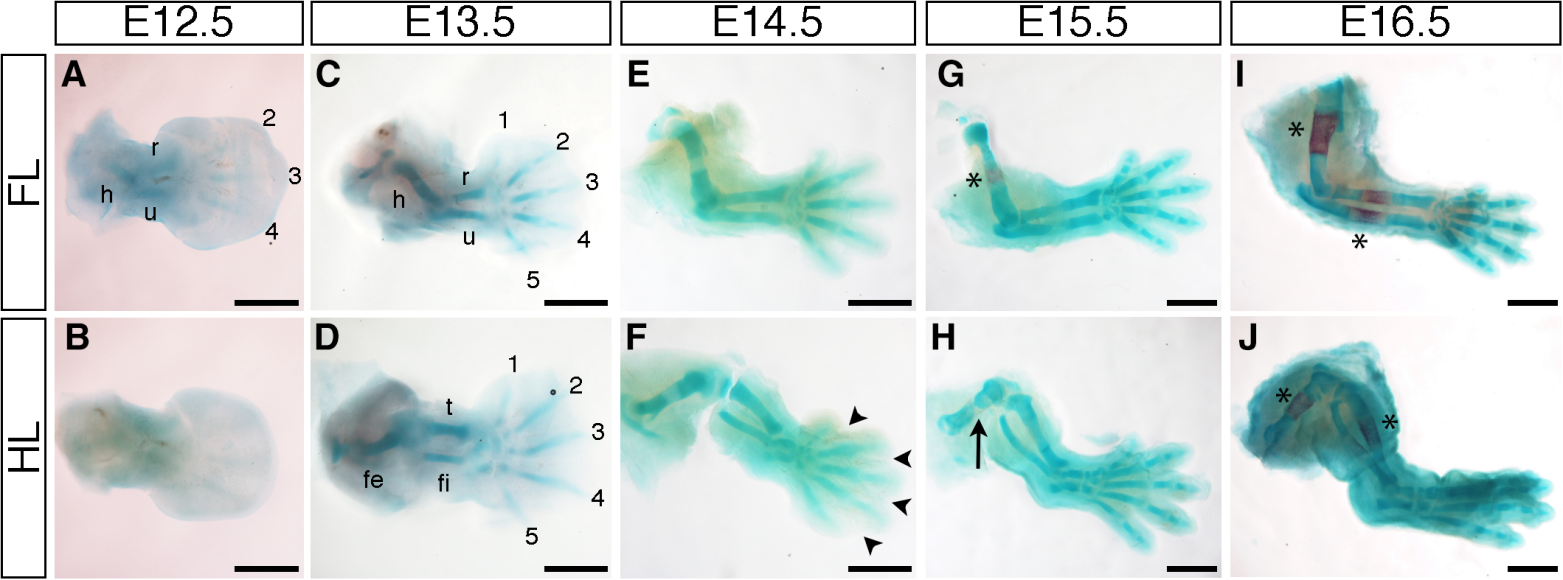
Cartilage and bone analysis. **A–J**: Staining with Alcian Blue and Alizarin Red to visualize onset and development of cartilage and bone in E12.5 (A,B), E13.5 (C,D), E14.5 (E,F), E15.5 (G,H), and E16.5 (I,J) forelimbs (A,C,E,G,I) and hindlimbs (B,D,F,H,J). Alcian Blue–stained cartilage condensations are observed in proximal forelimb tissue and in some distal digits from E12.5 (A). Like the nerve innervation patterns, the hindlimb lags behind the forelimb (B). All cartilage elements are seen in both forelimb and hindlimb by E13.5 (C,D), and elements in both limbs have lengthened considerably and look almost identical by E14.5, even though digit separation is yet to complete in the hindlimb (F arrowheads). Alizarin Red is first seen in the middle of the humerus (forelimb) and femur (hindlimb) at E15.5; staining is stronger and broader in the humerus (G asterisk) and only just detectable in a small part of the femur (H black arrow). By E16.5, Alizarin Red staining can be seen in the middle of all the long bones in both the forelimb and hindlimb (I,J, asterisk). h: humerus; r: radius; u: ulna; fe: femur; t: tibia; fi: fibula; 1: digit 1; 2: digit 2; 3: digit 3; 4: digit 4; 5: digit 5. Scale bars = 500 µm.

Formation of cartilage condensations and differentiation into bone was assessed in the mouse embryonic limb between E12.5 and E16.5 by Alcian Blue and Alizarin Red staining (Fig. 6). Alcian Blue stains cartilage and Alizarin Red stains differentiating bone. Alcian Blue staining was first observed in some forelimb elements at E12.5, namely the humerus, radius, ulna, and digits 2, 3, and 4 (Fig. 6A); the hindlimb had no obvious Alcian Blue staining (Fig. 6B). By E13.5, all cartilage elements in both forelimbs and hindlimbs (long bones and digits) were stained by Alcian Blue (Fig. 6C,D). Alizarin Red staining was not detected until E15.5, when it stained a region in the medial part of the humerus (Fig. 6G asterisk) and a very small domain in the medial part of the femur (Fig. 6H black arrow). By E16.5, all the long bones in both forelimb (humerus, radius, and ulna) and hindlimb (femur, tibia, and fibula) displayed strong region of Alizarin Red staining in the medial parts of the long bones (Fig. 6I,J black asterisks).

## Discussion

Previous research has focused on comparison of wild‐type expression patterns with mutant mouse models rather than on wild‐type expression alone through development. Analysis of limb development in mutant mice models requires comparison to a model of “normal” development, and this article provides a useful, informative resource for the research community to refer to when studying limb development of mutant mouse models. We have described expression of markers of a range of important tissues essential for limb development: *Gdf5, MyoD1*, and *Cdh5* from E13.5 to E15.5; *Scleraxis* from E12.5 to E15.5; and anti‐neurofilament marker from E10.5 to E14.5. Although previous expression patterns are published in part, we show the dynamic expression of these markers and the related tissue development throughout limb development. Expression patterns in fore‐ and hindlimbs are shown, along with section images and images of limb innervation through development.

Expression patterns analyzed are largely supported by previous publications. Meech et al., (2005) reported *Gdf5* expression in digit rays at E11.5 and in developing cartilage condensations by E13.5; however, we do not see *Gdf5* expression exclusively across developing joint sites until E14.5 (Meech et al., 2005; Ota et al., 2007; Seemann et al., 2005). This difference may be due to strain differences in embryonic development, which can exhibit differences in gestation periods (Degenkolbe et al., 2013; Huang et al., 2016). *Gdf5* is expressed across developing joint sites, including interphalangeal joints, from E14.5 (Chen et al., 2016; Houweling et al., 2001; Huang et al., 2016; Sohaskey et al., 2009). Our findings for *Scleraxis* expression are in agreement with previously published analyses of *Scleraxis*, indicating expression in the autopod and in developing digit rays and cartilage condensations at E12.5, as well as defined expression in developing tendons at E13.5 (Huang et al., 2015; McGlinn et al., 2005; Schweitzer et al., 2001) and along developing digits, with strongest expression across sites of developing interzones at E14.5 (Murchison et al., 2007; Schweitzer et al., 2001). *MyoD1* is expressed in muscle progenitors in the zeugopod, not the autopod, at E12.5. By E13.5, muscle blocks are visible in the zeugopod, with early expression visible in the autopod, supporting patterns observed in this study (Behrens et al., 2003; Hasson et al., 2010; L'honoré et al., 2010; Wood et al., 2013). Expression patterns of other blood vessel markers support the *Cdh5* expression patterns finding we have observed. Platelet endothelial cell adhesion molecule (PECAM; also known as CD‐31) is expressed strongly at the base of interdigital regions at E12.5 (Vieira et al., 2007), and anti‐CD31 staining is also seen along the periphery of developing digits, with expression strongest at the base of interdigital regions and at regions of developing interzones at E14.5 (Ben et al., 2012; Eshkar‐Oren et al., 2015). Nerve patterning of the limbs in our study is consistent with previous work, indicating innervation in the limbs from around E11.5 (Martin, 1990; Vieira et al., 2007), and nerve branching is seen in the autopod in both forelimb and hindlimb by E13.5, reaching to the base of prechondrogenic condensations (Vieira et al., 2007). Our work also shows that innervation occurs in the forelimb before the hindlimb, and the hindlimb lags behind in innervation extent until around E14.5 (Fig. 5).

Comparing the expression patterns between fore‐ and hindlimbs, patterning of *Gdf5* and *Scleraxis* is well established in the hand‐ and footplate by E13.5, and *MyoD1* expression is seen later in the hand‐ and footplate from E14.5. *Gdf5* expression is seen more distally than that of *Scleraxis* during limb development. At E14.5, *Gdf5* expression can be seen in the distal interphalangeal joints, but *Scleraxis* is seen only as distal as the proximal interphalangeal joints in expression domains that overlap with those of *Gdf5. Cdh5* expression is seen in the regions of developing digit cartilage from E13.5, and from E14.5 some stronger bands of expression can be seen across digits in regions that may also express *Gdf5* and *Scleraxis.*

Far later than the expression of *Cdh5, Gdf5*, and *Scleraxis*, the neural plexus enters the digit tips at E14.5 (Martin, 1990) (Fig. 5F–G`,N–O`). Nerves enter the limbs at E11.5, and progression is delayed behind the migration of myocytes into the limb (Martin, 1990) (Fig. 5). The delayed migration of nerves into the limb bud, behind that of migrating myocytes, is also reported in the developing chicken limb (Mahony et al., 2018). This suggests a conserved mechanism whereby nerves enter the limb after initial tissue outgrowth from the flank, possibly allowing for myocyte and cartilage differentiation in the aneural environment before rapid innervation proceeds. In agreement with our finding that limb patterning has already begun before nerves enter the limb, in chickens, the presence of nerves is not required for limb patterning (Harsum et al., 2001; Mahony et al., 2018; Strecker and Stephens, 1983). We used two assays to observe neuronal innervation into the mouse fore‐ and hindlimbs: immunofluorescence and immunoperoxidase (Fig. 5). Both assays clearly show nerve patterning in detail and largely complement each other, though it is also clear that there is a marked variability in staging of early limbs between litters: Some E11.5 limbs show some proximal innervation of hindlimbs, while embryos from other litters do not. This is likely due to differences of embryonic age within and between litters.

Skeletal patterning progresses in a proximal‐to‐distal manner, and once all the cartilage condensations (detected by Alcian Blue) have been patterned, they are gradually replaced by bone (detected by Alizarin Red) as limb development proceeds, again in a proximal‐to‐distal manner. The forelimb has cartilage condensations before the hindlimb in the humerus, radius, ulna, and middle digits (Fig. 6A,B). However, the hindlimb rapidly catches up and both limbs are difficult to differentiate by E12.5 (Fig. 6C,D). Alizarin Red staining is first seen in the proximal long bones (humerus and femur, respectively) before the medial long bones, again underlining that the patterning and differentiation of limb elements is in a proximal‐to‐distal manner (Fig. 6G–J).

In this article, we have described the expression patterns and developmental progression of markers of limb elements through the most active stages of mouse limb development. This array of expression patterns can be used to further understand normal mouse limb development and outgrowth, as well as to understand tissue changes and gene regulation following functional misexpression experiments.

## Experimental Procedures

### Mice

All experimental procedures and conditions were in accordance with institutional Animal Welfare and Ethical Review Bodies (AWERB) and UK Home Office guidelines. Wild‐type C57BL/6J mice were maintained in in‐house breeding colonies. Mice were mated and the morning of vaginal plug formation counted as E0.5. Pregnant mothers were killed by cervical dislocation and embryos fixed overnight in 4% paraformaldehyde (PFA) in phosphate buffered saline (PBS). Experimental protocols were each repeated at least twice, and the number of embryos used was a minimum of three on each occasion, and in most cases totaled between five and eight embryos per protocol.

### Whole‐mount In Situ Hybridization

To generate templates for the *MyoD1, Cdh5*, and *Gdf5* riboprobes, RNA was extracted from E14.5 and E15.5 mouse limbs using the Qiagen RNeasy Mini Kit and cDNA synthesized using SuperScript III (Thermo Fisher Scientific) according to the manufacturer's instructions. DNA fragments were isolated by Polymerase Chain Reaction (PCR) (94˚C for 5 min, 35 cycles of 94˚C for 40 sec, 55˚C for 1 min, 72˚C for 90 sec, followed by a final extension at 72˚C for 5 min) and cloned into either pGEM‐T Easy (Promega; *Gdf5*) or pBluescript KS + (*MyoD1* and *Cdh5*). The following primers were used: *Gdf5* forward, AAAGGGCAAGATGACCGAGG; reverse, ACGTTGTTGGCAGAGTCGAT; *MyoD1* forward, TTTTTGAGGACCTGGACCCG; reverse, TGCCATCAGAGCAGTTGGAG; *Cdh5* forward, TTGCCCTGAAGAACGAGGAC; reverse, GTCGGAGGAATTGGTGCTCA. The riboprobe template for *Scleraxis* was a gift from Dr. Ronen Schweitzer, Portland, Oregon. Antisense RNA probes were then prepared by taking plasmid DNA and using PCR with M13 forward and M13 reverse primers to generate the probe template. in vitro transcription with relevant RNA polymerase was then carried out to generate the riboprobe labeled with digoxigenin (we used a Boehringer Mannheim kit), followed by removal of unincorporated nucleotides by placing solution through a G‐50 spin column. Whole‐mount in situ hybridization was performed as follows: Embryos were dissected and fixed overnight in 4% PFA, dehydrated in 50% methanol in PBT (PBS with 0.1% tween), and then stored in 100% methanol after three times at ‐20C˚. Dehydrated embryos were bleached in 6% hydrogen peroxide in 100% methanol for 1 hr, then rehydrated via washes in 50% methanol:PBT and 25% methanol:PBT to PBT. Embryos (up to E12.5) were treated with 10 ug/ml of proteinase K for 20 min, E13.5 and E14.5 embryos were treated with proteinase K at 20 ug/ml for 15 min, and E15.5 embryos were treated with proteinase K at 40 ug/ml for 15 min. Embryos were rinsed in PBT and post‐fixed in 4% PFA for 20 min. Embryos were then incubated in prehybridization solution (50% formamide, 5X SSC pH4.5, 50 ug/ml yeast RNA, 1% SDS, 50 ug/ml heparin) at 70C˚ for 1 hr and then incubated in fresh prehybridization solution containing the relevant riboprobe that had been preheated to 85C˚ for 5 min prior to addition to the prehybridization mix (we use 10 ul of an in vitro transcription reaction per 1 ml of prehybridization mix). Hybridization was carried out overnight at 65˚C. Following hybridization, the probe can be removed and stored at ‐20C˚ for another in situ hybridization and reused up to five times before being remade. Embryos are then placed in solutions with varying stringency at 65C˚ to remove nondescript probe binding. First into solution 1 (50% formamide, 5X SSC, 1% SDS, pH4.5) for three washes of 30 min each, then into solution 3 (50% formamide, 5X SSC, pH4.5) for 3 washes of 30 min each, before being placed in TBST (tris‐buffered saline with 1% tween). Embryos are then blocked in a solution of 10% inactivated goat serum in TBST for 1 hr and then placed in the antibody solution of 1% inactivated goat serum in TBST containing anti‐DIG antibody at 1:5000 overnight at 4C˚. The following day, embryos are washed in TBST at least 10 times throughout the day. The next day, embryos are washed in NTMT (100 mM NaCl, 100 mM Tris‐HCl pH9.5; 50 mM MgCl_2_, 1% tween) three times (10 min each) and then placed in the color solution (1 ml NTMT with 4.5 ul BCIP [5’Bromo‐4‐chloro‐3‐indoly phosphate] (50 mg/ml) and 2.5 ul NBT [Nitroblue tetrazolium]; (5 mg/ml) at room temperature in the dark. Embryos were checked every 20 min, and the reaction was terminated when complete by washing in PBT, then post‐fixed in 4% PFA and stored in PBT.

### Immunofluorescence

Immunofluorescence staining was carried out based on previous protocols with some modifications (Kardon, 1998) using an anti‐neurofilament antibody (Millipore AB1987) and IgG goat anti‐rabbit Cy3 secondary antibody (Jackson ImmunoResearch). No antigen retrieval was carried out. Following overnight fixation in 4% PFA at 4˚C, embryos were washed in PBS and then placed in Dent's bleach (1 part hydrogen peroxide, 2 parts Dent's fix) overnight at 4˚C. Embryos were washed in 100% methanol and fixed in Dent's fixative (1 part Dimethyl sulfoxide, 4 parts methanol) overnight at 4˚C. After being rinsed multiple times in PBS, embryos were placed in the primary antibody in blocking solution overnight (18 hr minimum) at 4˚C. Following washes in PBS (over 6 hr), embryos were placed in the secondary antibody overnight at 4˚C. Embryos were then rinsed in PBS multiple times over a 6‐hr period, then placed in a 50% methanol:PBS solution, followed by 100% methanol washes, before being placed in BABB (33.3% benzyl alcohol, 66.6% benzyl benzoate) to clear and allow imaging under a fluorescence microscope.

### Immunoperoxidase Staining of Nerves

Immunoperoxidase staining was based on previously described protocols with modifications (Kardon, 1998) using an anti‐neurofilament antibody (Millipore AB1987) and a peroxidase conjugated secondary antibody (Sigma). Following overnight fixation in 4% PFA at 4˚C, embryos were washed in PBS and then placed in Dent's bleach overnight at 4˚C. Embryos were washed in methanol and fixed in Dent's fixative overnight at 4˚C. After being rinsed in PBS, embryos were blocked and then placed in the primary antibody for 2 days at 4˚C. Following multiple washes in PBS, embryos were placed in the secondary antibody overnight at 4˚C. Following PBS washes, embryos were incubated in fresh 3,3‐diaminobenzidine (DAB; Sigma) until reaction was complete, rinsed in methanol, and cleared and stored in BABB.

### Vibratome Sectioning

Embryos were embedded in 4% agarose in water and sectioned transversely using a Vibratome 1000 Plus at 100 µm. The sections were collected in 48 well plates containing PBS and mounted on Superfrost slides (VWR) with Vectashield mounting medium (Vector Labs).

### Alcian Blue and Alizarin Red Staining of Mouse Embryos

To observe cartilage and appearance of bone, we stained embryos with Alcian Blue and Alizarin Red using a protocol we modified from Rigueur and Lyons (2014).

Embryos were fixed in 70% ethanol overnight, placed into 95% ethanol for 1 hr, and incubated in acetone overnight at room temperature. Embryos were stained with 0.03% Alcian Blue for 4 hr, then with 0.005% Alizarin Red for 4 hr before being cleared in fresh 1% KOH for 1 hr (E11.5–E13.5) or 2 hr (E14.5–E16.5), then stored in glycerol.

### Imaging

Whole‐mount and section images were captured using a Nikon SMZ1500 microscope with Nikon DS‐L1 camera. Whole‐mount antibody stains were imaged using a Nikon SMZ1500 and a Nikon DXM1200 camera. Figures were prepared using Adobe Photoshop.
